# Influence of academic involution atmosphere on college students’ stress response: the chain mediating effect of relative deprivation and academic involution

**DOI:** 10.1186/s12889-024-18347-7

**Published:** 2024-03-21

**Authors:** Aichun Liu, Yanjin Shi, Yibo Zhao, Jianchao Ni

**Affiliations:** 1 Department of Physical and Health Education, Wuxi Vocational and Technical Institute of Commerce, Wuxi, 214153 Jiangsu China; 2 Institute of Education, University College London, London, WC1E 6BT UK; 3 Institute of Education, Xiamen University, Xiamen, 361005 Fujian China

**Keywords:** College students, Academic involution atmosphere, Relative deprivation, Academic involution, Stress response

## Abstract

**Background:**

In recent years, the phenomenon of academic involution atmosphere among college students has gradually attracted the focus of education and social circles. Thus, this study targets college students as the research object and constructs a hypothetical model to explore the relationship between academic involution atmosphere and college students’ stress response, as well as the mediating role of relative deprivation and academic involution.

**Methods:**

A survey was conducted on 1090 college students using the Academic Involution Atmosphere Scale, Relative Deprivation Scale, Personal Academic Involution Scale, and Stress Response Scale.

**Results:**

The results show that: (1) Academic involution atmosphere, relative deprivation, and academic involution are significantly and positively correlated with stress response; (2) Academic involution atmosphere not only directly predicts college students’ stress response, but also indirectly predicts them through relative deprivation and academic involution, respectively; (3) Relative deprivation and academic involution have a chain mediating effect between academic involution atmosphere and stress response.

**Conclusions:**

The findings of this study reveal the influence of academic involution atmosphere on college students’ stress response and the mechanism, providing beneficial insights for reducing college students’ stress response and maintaining their psychological well-being.

**Supplementary Information:**

The online version contains supplementary material available at 10.1186/s12889-024-18347-7.

## Introduction

With the increasingly fierce social competition, the phenomenon of academic involution atmosphere among college students has gradually become the focus of attention in educational and social realms. Academic involution refers to the immense pressure students undertake in order to achieve outstanding academic performance, often leading to excessive commitment to their studies at the expense of other aspects of development [1, 2]. This phenomenon not only negatively affects the physical and mental health of college students, but also has potential far-reaching influences on society. Academic involution atmosphere is an important part of academic involution phenomenon, representing a highly competitive and stressful learning environment. In this atmosphere, students often feel pressure from peers, family, and society [3], which, if not handled appropriately, may lead to serious physical and psychological problems. Stress response refers to is a persistent state of tension that occurs when external demands exceed individual’s capabilities and resources, which is an essential manifestation of individual’s mental health problems [4]. College students are regarded as the backbone of future social development, and their stress response is not only related to the achievement of academic goals, but also has an important impact on their future social adaptation and career development. It can be seen that it is of great value for the growth and development of college students and the quality of talents training in higher education to explore the stress response of college students and its influencing factors under the current academic involution atmosphere.

## Literature review and research hypotheses

### The influence of academic involution atmosphere on stress response

Among the environmental factors influencing college students’ stress response, academic involution atmosphere is a factor worth exploring. Recent years have witnessed a strong academic involution atmosphere permeating among Chinese college student [3], necessitating an examination of its impact on college students’ stress response. Academic involution atmosphere refers to the perception of students’ excessive commitment in their studies in order to obtain superior academic evaluations or educational resources in the school environment [3]. Although previous studies explored the influences of factors such as efficacy and coping strategies on stress responses [5, 6], few studies have investigated the effects of academic involution atmosphere on college students’ stress response. Based on External Stimulus Theory, environmental stimulus is regarded as stress [7]. As a prevalent environmental stimulus among Chinese college students in recent years, academic involution atmosphere leads students to perceive irrational and excessive over-competitive behaviors or phenomena, which undoubtedly triggers stress response of college students. In addition, according to Ecosystem Theory [8], the school represent an environment that college students are in direct contact with and a microsystem that influences them. Therefore, academic involution atmosphere in the school will inevitably impact college students’ stress response. Previous researchers have also suggested that students in a strong academic involution atmosphere may experience greater pressure to achieve good academic outcomes, which may increase negative emotions such as irritability and anxiety [3]. Thus, academic involution atmosphere is supposed to exert a positive influence on college students’ stress response. Current academia remain scarce empirical researches on the influence of academic involution atmosphere on stress response. Based on this, this study aims to explore the situation and proposes the hypothesis:

- H1: Academic involution atmosphere positively predicts college students’ stress response.

### The mediating effect of relative deprivation

How does academic involution atmosphere affect college students’ stress response? There is a lack of empirical research in current academia. Through literature review, this study suggests that relative deprivation might play a crucial role in this context [9]. Relative deprivation may play a mediating role in the process through which academic involution atmosphere influences college students’ stress response. Relative deprivation refers to individual’s or group’s perception of their disadvantaged status compared to a reference group [10]. This perceived disadvantage does not stem from absolute conditions of disadvantage but rather from the result of comparisons with others. Thus, relative deprivation is a product of social comparison and often involves comparing oneself with similar groups [11]. Firstly, academic involution atmosphere may positively influence college students’ relative deprivation. Classical Relative Deprivation Theory (RDT) suggests that individuals assess their status and situation primarily through comparisons with others [12]. College students in an academic involution atmosphere experience intense academic competition and comparisons, as well as escalated evaluation standards or thresholds for accessing educational resources, triggering relative deprivation. Secondly, relative deprivation exacerbates college students’ stress response. Social comparison is the core psychological process of relative deprivation [13], which encompasses not only horizontal comparisons between individuals or groups and reference groups, but also vertical comparisons between value expectation and value ability or between current situation and past or future condition. According to social comparison theory [14], individuals who compare themselves with others may increase their psychosocial stress when they perform unfavorably. That is, individual’s relative deprivation intensifies psychosocial stress, which is detrimental to their physical and mental health [15]. Existing researches have shown that individual’s relative deprivation and stress are closely related. Smith et al. [16] demonstrated that teachers who perceived that they were paid much less than their colleagues reported higher levels of stress. Callan et al. [17] found a significant correlation between individual’s relative deprivation and stress. Furthermore, Callan et al.‘s research [18] indicated that changes in individual’s relative deprivation remained significantly correlated with changes in stress levels over a six-week period. Based on the above analysis, this study proposes the hypothesis:

- H2: Academic involution atmosphere may influence stress response through the mediating effect of relative deprivation.

### The mediating effect of academic involution

In addition to relative deprivation, academic involution may also be a mediator between the academic involution atmosphere and stress response [19]. The term “involution” was initially proposed by American anthropologist Alexander Goldenweiser [20] to describe a situation where individuals engage in mutual competition and internal depletion for limited resources, which is similar to “vicious competition” and results in a continual decrease in individual’s “gain-to-effort ratio” [21]. In recent years, this concept has been widely applied in the field of education, generally referring to students’ over-commitment to irrational competition in pursuit of limited educational resources or opportunities [1, 2]. Firstly, academic involution atmosphere positively predicts academic involution. In this atmosphere, college students have a strong sense of academic competition, striving to enhance their competitive advantage by excessively investing in their studies, thus leading to behaviors of academic involution [22].

Secondly, higher levels of personal academic involution correspond to higher stress response. Academic involution is essentially a competition-driven behavior of over-commitment in studies [23]. On the one hand, it may help students achieve good academic performance. On the other hand, it may also bring them substantial academic pressure. Previous studies [19, 24] have found a significant positive correlation between academic involution in education and individuals’ anxiety and stress. Academic involution exacerbates competitive pressures among students, increasing their perception of stress [25]. Researches also indicated that intense academic competition often triggers excessive concern about academic performance, accompanied by corresponding psychological pressures, insecurity, and anxiety [26]. In other words, college students’ personal academic involution may subject them to greater stress and exacerbate stress response. Based on this, the study proposes the hypothesis:

- H3: Personal academic involution mediates the relationship between the academic involution atmosphere and stress response.

### The chain mediating effect of relative deprivation and academic involution

In addition to exploring the mediating role of relative deprivation and academic involution in the relationship between the academic involution atmosphere and stress response, this study also investigates the correlation between relative deprivation and academic involution. Existing studies lack exploration into the relationship between relative deprivation and academic involution. According to the intrinsic characteristics of relative deprivation and academic involution, this study infers that relative deprivation may have a positive influence on academic involution. In the academic involution atmosphere, students experience intense academic competition and comparisons, as well as elevated evaluation standards or thresholds for obtaining educational resources, consequently leading to a sense of relative deprivation [27]. Students with high academic expectations are more likely to experience relative deprivation, thereby displaying stronger tendencies toward academic involution [28]. The core psychological process of relative deprivation is social comparison [29], which often generates competition. Academic involution represents intense competitive behavior of over-commitment driven by comparison [30]. Thus, relative deprivation may positively predict academic involution. Based on the above analysis, the study proposes the hypothesis:

- H4: Relative deprivation and academic involution may play a chain mediating effect between academic involution atmosphere and stress response.

**Fig. 1:**
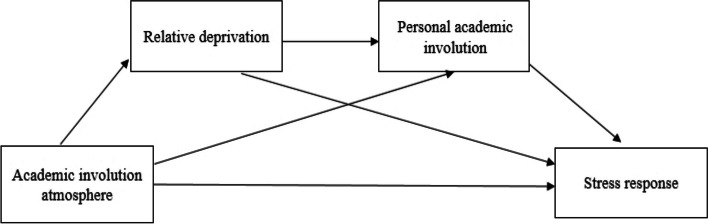
The chain mediating model of relative deprivation and academic involution

In summary, academic involution atmosphere, relative deprivation, academic involution, and stress response are closely related. Currently, no study has yet examined the combined mechanisms of academic involution atmosphere, relative deprivation, and academic involution on stress response. Drawing on previous theories and researches, this study targets college students as the research object and constructs a hypothetical model as shown in Fig. 1, with the purpose of exploring the underlying mechanisms of college students’ stress response in the academic involution atmosphere. It analyzes how academic involution atmosphere influences stress response and the chain mediating effect of relative deprivation and academic involution. It aims to better understand and address the pressure faced by current college students, provide a theoretical basis for alleviating college students’ stress response, and consequently offer more targeted suggestions and strategies for their mental health education.

## Methodology

### Data sources and sample characteristics

On the basis of ensuring the scientific design of the survey, and taking into account the principles of feasibility and cost-effectiveness of the design, this study adopts the convenience sampling method, which is fast, simple, easy to obtain and cost-effective. In this study, six colleges - Xiamen University, Quanzhou Normal University, Jilin University of Finance and Economics, Nanjing Tech University Pujiang Institute, Wuxi Vocational and Technical Institute of Commerce, and Nanjing institute of tourism and hospitality were selected as universities of different levels, with a total of 1,200 college students to conduct the questionnaire survey. The sample covered both undergraduate and vocational colleges and universities, with a wide geographical distribution across the south, central and north of China. The questionnaire was mainly paper-based, supplemented by electronic version, and data were collected simultaneously through a combination of online and offline questionnaires. The online questionnaire was sent to the target sample via the Questionnaire Star website (https://www.wjx.cn/) with a link to invite them to fill in. Meanwhile, in order to reach those who preferred paper questionnaires, we also designed and printed paper versions of the questionnaires, which were distributed and collected in classrooms, dormitories, and other specific locations. In order to protect the privacy of participants and ensure data anonymity, questions that could be directly associated to personal identity, such as name, address, or other information about personal identifiers, were avoided during the phase of questionnaire design. For online questionnaires, the system automatically processed them as anonymous submissions, and participants were not required to register or log in when completing the questionnaire, thus avoiding the possibility of direct tracing. For paper-based questionnaires, the data were also anonymized during data collation and entry. Personal information was separated from actual responses and only coded data were retained for subsequent analysis.

### Research tools

#### Academic involution atmosphere scale

The College Academic Involution Atmosphere Scale developed by [3] was used, which consists of 6 items. For example, “I feel that most classmates compete fiercely in academics” and “I feel that most classmates put in excessive effort in their studies”. It primarily measures college students’ perception about the situation that the majority in the university environment excessively invest in their academics to obtain superior teaching evaluations or educational resources. It is designed to reflect the degree of the majority’s academic involution in the environment perceived by individuals. A 5-point Likert scale was used, with higher scores indicating a higher degree of agreement with the given statements. The KMO value is 0.912, indicating the research data are suitable for extracting information; the Cronbach’αis 0.934, signifying favorable consistency and valid measurement results. The items were summed and averaged to derive the variable of academic involution atmosphere. The higher scores, the higher degrees of academic involution atmosphere.

#### Stress response scale

With reference to Student-Life Stress Inventory developed by Gadzella [31] and Mental Disorders Scale developed by Spitzer et al. [32], the questionnaire items were selected and adjusted according to the actual situation of college students. The scale consisting of 8 items primarily measures the persistent physical and mental tension of college students, aiming to reflect the degree of stress perceived by them. For example, “I feel nervous and anxious easily” and “I often feel depressed and in low spirits”. A Likert 5-point scale was used, with higher scores indicating a higher degree of agreement with the questions. The KMO value is 0.936, indicating the research data are suitable for extracting information; the Cronbach’αis 0.944, signifying favourable consistency and valid measurement results. The items were summed and averaged to derive the variable of stress response. The higher scores, the higher degrees of stress response.

#### Relative deprivation scale

Relative Deprivation Questionnaire developed by Ma [33] was used, comprising 4 items. For example, “Compared with the efforts and contributions I have made, my life should be better than it is now” and “I always feel that others have taken possession of what should belong to me”. It mainly measures the level of relative deprivation felt by individuals, and is designed to reflect the level of relative deprivation. A 5-point Likert scale was used, with higher scores representing higher degrees of agreement with the given statements. The KMO value is 0.779, indicating the research data are suitable for extracting information; the Cronbach’αis 0.824, signifying favourable consistency and valid measurement results. The items were summed and averaged to derive the variable of relative deprivation. The higher scores, the stronger relative deprivation.

#### Personal academic involution scale

Personal Academic Involution Scale developed by Zhou Xiting et al. [3] was used and adjusted according to the actual situations of college students. Consisting of 6 items, it primarily measures typical academic involution behaviors in the university environment, aiming to reflect the degree of college students’ academic involvement. For example, “I often compete fiercely with my classmates in academics” and “I often put in excessive effort in my studies”. A 5-point Likert scale was used, with higher scores representing higher degrees of agreement with the questions. The KMO value is 0.879, indicating the research data are suitable for extracting information; the Cronbach’αis 0.871, suggesting favourable consistency and valid measurement results. The items were summed and averaged to derive the variable of personal academic involution. The higher scores, the fiercer personal academic involution.

### Data analysis

The self-report method of data collection may bring about the common method bias. The Harman’s single-factor test was employed to examine this issue. The results showed that there are four factors with eigenvalues greater than 1. The total variance explained by the first common factor is 36.682% (Table 2), which is less than the critical value of 40%. Therefore, the research data does not have the common method bias problem. Additionally, all variables were standardized beforehand to avoid bias in moderating effects. Subsequently, descriptive statistics and the Pearson correlation analyses of the main variables were conducted by using SPSS 26. Generally speaking, a correlation coefficient *r* < 0.3 indicates a weak linear relationship between two variables. The correlation coefficient r is between 0.3 and 0.7, indicating a certain degree of linear relationship between the two variables [34]. Process plugin by Hayes [35] was used to analyze mediating and moderating effects. The significance of the mediating effect was examined using the bias-corrected Bootstrap method. It is considered statistically significant if the 95% confidence interval does not include zero. In addition, all variables were standardized beforehand to avoid bias in the moderating effect. The chain mediating test was used to estimate whether the independent variable had an effect on the dependent variable through multiple mediating variables. During the test, multiple regression analysis is commonly used to determine the correlation between each mediating variable and the dependent variable, and the chain mediating effect is verified by calculating the indirect effect [36]. The meaning of the regression coefficient is a measure of the degree of influence of the independent variable on the dependent variable, also known as the slope. In regression analysis, the regression coefficient is commonly used to evaluate the relationship between independent and dependent variables. In this research, Model 6 of the process plugin developed by Hayes was used to estimate the chain mediating effect of relative deprivation and personal academic involution in the relationship between the academic involution atmosphere and individual academic involution. The independent variable in this study is academic involution atmosphere, the dependent variable is stress response, and the mediating variables are relative deprivation and personal academic involution. The 95% confidence interval (CI) for the mediating effect was estimated by extracting 5,000 bootstrap samples. Significance of the effects is tested by whether the CI contains 0. Non-inclusion of zero in the CI indicates a significant effect.

**Table 1:** Initial eigenvalues

Ingredient	Total	Percentage of variance	Cumulative %
1	8.804	36.682	36.682
2	4.372	18.219	54.900
3	2.413	10.052	64.953
4	1.153	4.803	69.756

## Research results

### Sample characteristics

1,200 questionnaires were sorted and 110 invalid questionnaires were excluded (the exclusion criteria: answering time less than 2 min, inconsistent logic in responses, many questions skipped or missed, etc.). The effective response rate of the questionnaire is 90.83%. The introductory part of the questionnaire contains sociodemographic variables such as gender, residence, family upbringing modes, and famil financial situation so that readers can understand the characteristics of the sample. As seen from Table 1, the sample is representative, for its relatively balanced distribution in demographic variables, which could to some extent mitigate the influence of the sample on the results (Table 2).

**Table 2:** Basic characteristics of the sample (*N* = 1090)

Variable name		Frequency	Percentage
Gender	Male	543	49.8
	Female	547	50.2
Residence	Rural	630	57.8
	Urban	460	42.2
Family upbringing modes	authoritative	215	19.7
	autocratic	254	23.3
	indulgent	541	49.6
	neglectful	80	7.3
Family financial situation	straitened	174	16.0
	average	829	76.1
	relatively good	87	8.0

### Correlation analysis of the key variables

The results of Pearson correlation analyses revealed a significant positive correlation among academic involution atmosphere, stress response, relative deprivation, and personal academic involution (Table 3). Where, Relative deprivation and Stress response (*r* = 0.645, *p* < 0.01), Personal academic involution and Academic involution atmosphere (*r* = 0.417, *p* < 0.01), Relative deprivation and Academic involution atmosphere (*r* = 0.343, *p* < 0.01) are well correlated, while the correlation among other variables is weak, but still statistically significant.

**Table 3:** Descriptive statistics and correlation matrix of the variables

Variables	M	SD	1	2	3	4
1 Academic involution atmosphere	2.98	0.77	1			
2 Stress response	2.59	0.90	0.291**	1		
3 Relative deprivation	2.56	0.78	0.343**	0.645**	1	
4 Personal academic involution	2.66	0.69	0.417**	0.159**	0.299**	1

* means *p* < 0.05, ** means *p* < 0.01, *** means *p* < 0.001

### Hypothetical model testing

**Fig. 2:**
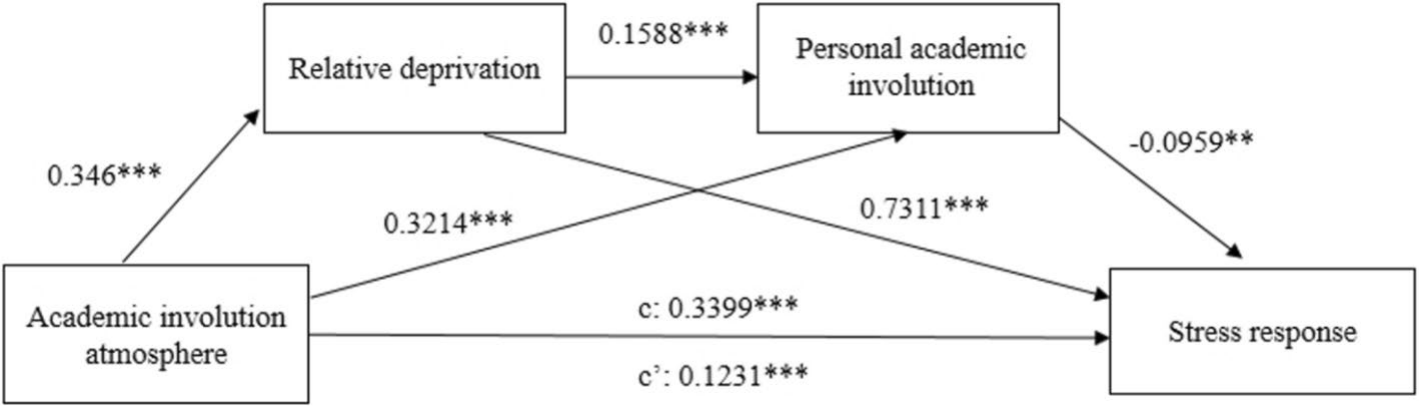
The chain mediating test model

The mediating effect model is primarily used to explore how one or more variables mediate between the independent and dependent variables, influencing their relationship. A chain mediating effect model (Model 6) is used in this study for hypothesis testing. Tables 4 and 5, and Fig. 2 show the results of the chain mediating test model.

**Table 4:** The chain mediating regression equation

Outcome variable	Predictor variable	*R*^2^	F	Std.Error	β	t
Relative deprivation	Constant	0.1173	144.6156***	0.7305	1.5251***	17.2378
	Academic involution atmosphere				0.346***	12.0256
Personal academic involution	Constant	0.2014	137.1075***	0.6216	1.2935***	15.2272
	Academic involution atmosphere				0.3214***	12.3349
	Relative deprivation				0.1588***	6.155
Stress response	Constant	0.0845	100.3923***	0.8615	1.5736***	15.0809
	Academic involution atmosphere				0.3399***	10.0196
Stress response	Constant	0.4260	268.8749***	0.6826	0.6059***	5.8961
	Academic involution atmosphere				0.1231***	4.0296
	relative deprivation				0.7311***	25.3669
	Personal academic involution				-0.0959**	-2.8801

* means *p* < 0.05, ** means *p* < 0.01, *** means *p* < 0.001

The results show that academic involution atmosphere positively and significantly predicts stress response, supporting Hypothesis 1. The 95% CI of the total effect of academic involution atmosphere on stress response is (0.2734, 0.4065), excluding 0, signifying a significant total effect. After introducing the mediating variables of relative deprivation and personal academic involution, the chain mediating model without control variables (Model1) is established. The direct effect of academic involution atmosphere on stress response remains significant, with the 95% CI of (0.0632, 0.1831). The chain mediating effect (accounting for 63.78% of the total effect) mainly consists of the following three pathways (Table 5):

1. Academic involution atmosphere -> Relative deprivation -> Stress response [95% CI = (0.2007, 0.3055), excluding 0], Hypothesis 2 holds;
2. Academic involution atmosphere -> Personal academic involution -> Stress response [95% CI = (-0.0568, -0.0069), excluding 0], Hypothesis 3 holds;
3. Academic involution atmosphere -> Relative deprivation -> Personal academic involution -> Stress response [95% CI = (-0.0104, -0.0012), excluding 0], Hypothesis 4 holds.

**Table 5:** Bootstrap test for mediating effect (Model1)

Model1	Effect	Boot SE	Boot LLCI	Boot ULCI
Total effect	0.3399	0.0339	0.2734	0.4065
Direct effect	0.1231	0.0306	0.0632	0.1831
Total mediating effect	0.2168	0.0294	0.1588	0.2737
Ind1	0.2529	0.0269	0.2007	0.3055
Ind2	-0.0308	0.0126	-0.0568	-0.0069
Ind3	-0.0053	0.0023	-0.0104	-0.0012

Ind1: Academic involution atmosphere -> Relative deprivation -> Stress response

Ind2: Academic involution atmosphere -> Personal academic involution -> Stress response

Ind3: Academic involution atmosphere -> Relative deprivation -> Personal academic involution -> Stress response

Boot SE: Bootstrap Standard Error, the standard error of the indirect effect estimate obtained by the Bootstrap sampling method

Boot LLCI: Bootstrap Lower Limit of the Confidence Interval, which represents the minimum estimated value that the true value of the indirect effect may fall into at a certain confidence level

Boot ULCI: Bootstrap Upper Limit of the Confidence Interval, which represents the maximum estimated value that the true value of the indirect effect may fall into at a certain confidence level

**Table 6:** Bootstrap test for mediating effect (Model2)

Model2	Effect	Boot SE	Boot LLCI	Boot ULCI
Total effect	0.3452	0.0338	0.2788	0.4116
Direct effect	0.1211	0.0309	0.0605	0.1818
Total mediating effect	0.2241	0.0288	0.1683	0.2816
Ind1	0.2581	0.0264	0.2082	0.3116
Ind2	-0.029	0.0132	-0.0559	-0.0049
Ind3	-0.005	0.0025	-0.0105	-0.0008

Sensitivity analysis is a statistical tool designed to assess the robustness and sensitivity of a model or system to adjustments of key parameters. The process involves trying to include or exclude specific variables in the model construction and re-estimating the model parameters to test the extent to which they affect the overall conclusion. This study mainly compares the changes of mediating effect parameters when control variables are not introduced (Model1) and after control variables are introduced (Model2). Specifically, a chain mediation model (Model2) is constructed that included control variables such as gender, residence, family upbringing modes, family financial situation. Through comparative data analysis of Model1 (Table 5) and Model2 (Table 6), we find that the chain mediating effect ratio calculated by Model2 is 64.91%, which only changes slightly compared with 63.78% of Model1. Based on this, the main parameters of the mediating effect remain relatively stable after the inclusion of control variables, which indicates that the model has a good robustness to the inclusion of control variables.

## Discussion

Based on previous theoretical analyses and research results, this study targets college students and proposes a mechanism model for the influence of academic involution atmosphere on college students’ stress response. The results reveal a significant positive correlation between academic involution atmosphere and college students’ stress response, and relative deprivation and academic involution play a chain mediating role between academic involution atmosphere and stress response.

### The influence of academic involution atmosphere on stress response

The results of this study demonstrate a significant positive correlation between academic involution atmosphere and college students’ stress response. Academic involution atmosphere positively predicts stress response, which is consistent with the existing research [3]. According to External Stimulus Theory [7], environmental stimuli equate to stress. In the academic involution atmosphere, college students face intense competitive pressure. Due to limited resources such as grades, scholarships, internship, and job opportunities, college students often need to excel in all aspects to obtain better opportunities. This kind of competitive pressure leads college students to a long-lasting state of tension and anxiety, thus impacting their physical and mental health. In addition, according to Ecosystem Theory [8], the school environment is a microsystem that affects college students. Academic involution atmosphere in universities often compels students to deal with a large number of academic tasks and extracurricular activities. They may need to complete substantial assignments, prepare for exams, participate in club activities, etc. within a limited time. The time-management pressure can cause them to feel anxious and strained, and experience severe stress response.

### The mediating effect of relative deprivation

The study found that relative deprivation mediates the relationship between academic involution atmosphere and stress response. As the classical Relative Deprivation Theory (RDT) suggests, individuals evaluate their status and situation primarily via comparisons with others [12]. In an academic involution atmosphere, college students may be more inclined to compare themselves with others to assess their own status and value. When they feel themselves disadvantaged in certain aspects, relative deprivation arises and triggers stress response [15]. On the other hand, according to Social Comparison Theory [14], individuals who compare themselves with others may exprience increasing socio-psychological stress when they perform poorly. In an academic involution atmosphere, college students often engage themselves in comparisons with their peers. They may pay attention to the advantages possessed by others in academic performance, awards, internship opportunities, etc. and compare themselves to them. The perception of being inferior compared to others in some aspects generates stress response [17]. Besides, in the academic involution atmosphere, resources such as scholarships, internship, and job opportunities are limited. When students realize the scarcity of these resources and believe they are not able to attain opportunities equal to others, they experience relative deprivation. This sense of relative deprivation intensifies their stress response as they may worry about limitations in their future prospects. In essence, the stronger the academic involution atmosphere, the greater relative deprivation felt by college students, thus inducing more severe stress response.

### The mediating effect of academic involution

This study also found that academic involution has a mediating effect between academic involution atmosphere and stress response. In an academic involution atmosphere that emphasizes competition, college students often develop a strong sense of academic competitiveness. They try various ways to increase their academic commitment, in order to improve the chances of winning in the fierce competition. This tendency leads to their over-commitment in academics, resulting in the behavior of academic involution. Students’ objectives are often closely linked to grades and rankings. They not only need to maintain academic excellence, but may also need to participate in various extracurricular activities, competitions, etc., to increase their competitiveness. This sustained competitive pressure may give rise to physical and mental exhaustion, generating stress response [23]. In addition, academic involution means that students need to spend more time and effort in completing academic tasks while participating in many extracurricular activities and competitions. The heavy academic load can make students stressed because they need to constantly improve their grades and performance. Lastly, apart from the heavy academic load, students also worry about whether they are good enough to outperform others, which necessitates continuous efforts to maintain a top position. The increased pressure exacerbates their stress response [25]. Therefore, a strong academic involution atmosphere intensifies the degree of academic involution, causing more severe stress response of college students.

### The chain mediating effect of relative deprivation and academic involution

The last finding of the study shows that relative deprivation and academic involution have a chain mediating effect between academic involution atmosphere and stress response. In an academic involution atmosphere, college students experience intense academic competition [27]. Compared to others, students may feel that they are not adequate in academic achievements, resources, or opportunities, or they do not study hard enough, all of which will give them a sense of relative deprivation. Once students experience relative deprivation, they may spend more effort in studying to enhance their competitiveness, thereby increasing the degree of academic involution [30]. However, this could potentially escalate their stress response. On one hand, excessive academic commitment can make college students feel physically and mentally exhausted and cause uncomfortable symptoms such as headaches, insomnia, etc. On the other hand, they might excessively focus on grades and rankings, demanding too much from their own performance. It will produce negative emotions like self-blame, anxiety, and low self-esteem. As a result, academic involution atmosphere induces college students’ relative deprivation, which subsequently increases stress response through excessive studying. In other words, academic involution atmosphere influences college students’ stress response by generating relative deprivation through academic involution. This influential mechanism may further exacerbate students’ psychological and physical health problems.

### Research implications and limitations

This study explored the mechanism through which academic involution atmosphere influences stress response. It suggests that in an overly competitive academic environment, college students may feel incapable of keeping up with their peers and experience relative deprivation as a result. This feeling drives them to continually strive to strengthen their competitiveness, but it also brings about physical and psychological stress. This research helps us better understand the influence of academic involution atmosphere on stress response, theoretically confirming and further enriching External Stimulus Theory [7], Ecological Systems Theory [8], Relative Deprivation Theory [12], and Social Comparison Theory [14]. Meanwhile, the findings of this study have significant implications for educational practices for college students. Firstly, universities should advocate a more inclusive, balanced, and comprehensive educational philosophy, encouraging diverse development and evaluation methods rather than merely pursuing academic performance. This shift could reduce unnecessary excessive competition and stress, enabling students to learn and grow in a relaxing and enjoyable atmosphere. Secondly, college students need to learn to adjust their mindset and expectations, focusing on personal growth and progress rather than excessive comparisons with others. At the same time, they should also learn to cope with academic pressure reasonably and seek necessary support and assistance to maintain a healthy lifestyle—both physically and psychologically. Thirdly, universities should prioritize the psychological well-being of college students, providing appropriate guidance and support to help them confront academic and life pressure. Fourth, promote the implementation of a flexible academic system that allows students to freely choose courses within a certain range to suit their individual learning paces and interests. This can help alleviate academic stress and motivate students to engage more actively in their studies. Fifth, develop policies to encourage students to engage in extracurricular activities such as sports, arts, and volunteer services. This approach aids in fostering the all-round development of students and reducing their excessive anxiety over purely academic achievements. Sixth, a closer home-school cooperation mechanism could be established to jointly focus on both students’ academic and mental health through communication with parents. Home-school cooperation helps create a more supportive environment for students’ development.

Despite new findings, certain limitations of this study exist that necessitate improvement and refinement in future research. Firstly, this research is a cross-sectional study, making it unable to ascertain the causal relationship between variables. Future studies could attempt to intervene via experiments or conduct longitudinal tracking to investigate whether the relationships between the academic involution atmosphere, relative deprivation, academic involution, and stress response change over time. Secondly, the variables in this study were self-reported by students, potentially leading to subjectivity and measurement biases. To mitigate such biases, future studies could assess variables based on reports from multiple subjects, thereby improving the accuracy and reliability of the measurement. Thirdly, there may exist not only one-way predictive relationships but also bidirectional predictive relationships among the four variables: academic involution atmosphere, relative deprivation, academic involution, and stress response, which remains to be further revealed by future studies. For example, academic involution might in turn affect the academic involution atmosphere, as increased each individual academic involution behavior inevitably intensifies the overall atmosphere. Fourth, this study explores the influence mechanism model of academic involution atmosphere on college students’ stress response. There may exist other mediating variables in this model, such as academic self-handicapping, rumination, etc., which could be analyzed in future research. Fifth, this study adopted a convenience sampling method, which may lead to selection bias, which may limit the generalizability of the research results to certain extent. Further research could implement stratified random sampling and expand the sample size to enhance the generalizability of the research results.

## Conclusion

Academic involution atmosphere can not only predict the stress response of college students directly, but also positively predict the stress response through the relative sense of deprivation, and can intensify the stress response through the chain mediating effect between relative deprivation and academic involution.

### Supplementary Information

**Supplementary Material 1.**

## Data Availability

The data that support the findings of this study are available from the corresponding author upon reasonable request.
