# Supplementary material for: Influence of academic involution atmosphere on college students’ stress response: the chain mediating effect of relative deprivation and academic involution
Source: BMC Public Health. 2024 Mar 21;24:870. doi: 10.1186/s12889-024-18347-7 (PMC10956225; doi:10.1186/s12889-024-18347-7)
Supplement: Supplementary file 1 — Supplementary Material 1. [file 12889_2024_18347_MOESM1_ESM.docx]

Appendix

Academic Involution Atmosphere Scale

1. I feel that most classmates compete fiercely in academics.

2. I feel that most classmates put in excessive effort in their studies

3. I feel that most classmates are trying to increase the number of words in course papers, experiment reports, etc. to achieve high scores.

4. I feel that most classmates intend to perform well in front of the teacher in order to get high scores in the course.

5. In order to obtain high scores, I feel that most classmates often complete tasks beyond the course requirements.

6. In order to obtain high scores, most classmates around me put in unlimited effort.

Stress Response Scale

1. I feel nervous and anxious easily.

2. I often feel depressed and in low spirits.

3. I feel scared for no reason.

4. I get upset or feel frightened easily.

5. I often feel weak and tired.

6. I often have difficulty breathing.

7. My heart often beats very fast.

8. I have poor sleep at night.

Relative Deprivation Scale

1. Compared with the efforts and contributions I have made, my life should be better than it is now.

2. I always feel that others have taken possession of what should belong to me.

3. Compared with people around me, I am disadvantageous in various aspects, life, study, work, etc.

4.Most of the rich people in the society make a fortune through dishonorable means.

Personal Academic Involution Scale

1. I often compete fiercely with my classmates in academics.

2. I often put in excessive effort in my studies.

3. I try to increase the word count of my course papers, experiment reports, etc. in order to get high scores.

4. I intentionally try to perform well in front of the teacher in order to get high scores in the course.

5. I often go beyond the requirements of the course in order to get high scores.

6. In order to get high scores, I put in my effort without limits.
